# Transmissive Labyrinthine Acoustic Metamaterial‐Based Holography for Extraordinary Energy Harvesting

**DOI:** 10.1002/adem.202201117

**Published:** 2022-11-09

**Authors:** Shubhi Bansal, Christabel Choi, James Hardwick, Biswajoy Bagchi, Manish K. Tiwari, Sriram Subramanian

**Affiliations:** ^1^ Department of Computer Science Faculty of Engineering University College London London WC1E 6BT UK; ^2^ Wellcome/EPSRC Centre for Interventional and Surgical Sciences University College London London W1W 7TS UK; ^3^ Nanoengineered Systems Laboratory Mechanical Engineering University College London London WC1E 7JE UK

**Keywords:** acoustic, energy, harvesting, metamaterials, piezoelectric

## Abstract

Conventional energy sources are continuously depleting, and the world is actively seeking new green and efficient energy solutions. Enormous amounts of acoustic energy are dissipated daily, but the low intensity and limited efficiency of current harvesting techniques are preventing its adoption as a ubiquitous method of power generation. Herein, a strategic solution to increase acoustic energy harvesting efficiency using a specially designed metamaterial is implemented. A scalable transmissive labyrinthine acoustic metamaterial (LAM) is designed, developed, and employed to maximize ultrasound (40 kHz) capture over its large surface area (>27 k mm^2^), which is focused onto a piezoelectric film (78.6 mm^2^), thus magnifying incident sound pressure by 13.6 times. Three different piezoelectric films – two commercial and one lab‐made nanocomposite film are tested with LAM in the acoustic energy harvesting system. An extraordinary voltage gain of 157–173% and a maximum power gain of 272% using the LAM compared to the case without the LAM are achieved. Multipoint focusing using holographic techniques, showcasing acoustic patterning to allow on‐demand simultaneous harvesting in separate locations, is demonstrated. Our versatile approach for high‐intensity acoustic energy harvesting opens future opportunities to exploit sound energy as a resource to contribute toward global sustainability.

## Graphical abstract

Herein, a novel approach to increase the efficiency of acoustic energy harvesting systems using a labyrinthine acoustic metamaterial is presented. The metamaterial captures acoustic energy from the sound source and focuses on the output sound onto a harvester. Ultrasound harvesting is experimentally demonstrated with three different piezoelectric films, and an extraordinary 272% maximum power gain is achieved using the metamaterial.

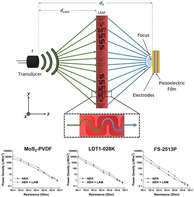

## 1 Introduction

Renewable energy sources^[^ ^1^ ^]^ like solar, wind, geothermal, and hydroelectric have become widely adopted in recent years, but one energy resource provided by myriad modern systems has still been underutilized – that is “sound.” Although the sound is in endless supply, it is mostly ignored and wasted due to the lack of appropriate harvesting technology and infrastructure. A promising candidate to address this problem would be acoustic metamaterial (AMM) technology. AMMs are artificially engineered materials that provide exceptional control for directing, manipulating, and mitigating sound waves across a broad frequency spectrum up to the megahertz range.^[^ ^2^, ^3^ ^]^ Recently, AMM‐based acoustic energy harvesting (AEH) has been explored via resonance‐based designs,^[^ ^4^ ^]^ often using Helmholtz resonator structures.^[^ ^5^, ^6^ ^]^ These include tapered neck resonators,^[^ ^7^ ^]^ planar resonators,^[^ ^8^ ^]^ and enhanced quarter spherical acoustic energy harvesters based on dual Helmholtz resonators.^[^ ^9^ ^]^ On a larger scale, acoustoelastic “MetaWall” noise barriers^[^ ^10^ ^]^ have been developed for industrial applications with simultaneous AEH. Similarly, elastic metastructures like phononic crystals (PCs)^[^ ^11^ ^]^ have demonstrated energy harvesting, with common structures like gradient index (GRIN) and topological devices.^[^ ^12^ ^]^ However, both often operate with surface acoustic waves, which can also go up to the high ultrasonic mega‐hertz range. For bulk acoustic waves, GRIN Luneburg lenses are capable of reflection, transmissive beam formation, and flexural wave collimation, making them well suited for antenna applications.^[^ ^13^, ^14^ ^]^ To implement energy harvesting with both surface and bulk acoustic waves, piezoelectric harvesters are directly integrated with the PC at the surface of the plate or attached at the output, with power output in the range of micro‐watts to milli‐watts.^[^ ^15^, ^16^ ^]^ However, these methods for both AMMs and PCs are inefficient because the impinging sound waves received by the harvester are of low intensity unless the sound source is in direct contact or in proximity to the harvester.

In the environment, acoustic energy has a small energy density. Two main strategies can be employed to enhance harvesting efficiency. One strategy is by focusing the acoustic energy onto a relatively smaller region where a receiver is placed. Previously, a harvesting mechanism through acoustic focusing was numerically simulated, where a multilateral reflective metamaterial created an enclosed system that reflected sound energy onto a piezoelectric bimorph situated behind the sound source.^[^ ^17^ ^]^ The system was limited for practical implementation as the location of the sound source was restricted within the enclosure, but it demonstrated how focusing can powerfully amplify the captured energy and reduce losses. The other strategy is to ensure that the receiver end captures a greater amount of acoustic energy by increasing the receiver area, for instance, by placing a higher number of low‐cost receivers. This concept is demonstrated by Xue et al.,^[^ ^18^ ^]^ where irradiation cross‐linked polypropylene (IXPP) piezoelectret films increased the active harvesting area and power output as they are larger than conventional polyvinylidene fluoride (PVDF).

Complex spatial distributions of sound pressure can be generated by holographic AMMs^[^ ^19^, ^20^, ^21^, ^22^ ^]^ through amplitude and phase modulation. Such AMMs enjoy a high degree of flexibility, where any desired image can be reconstructed and projected with high resolution down to the submillimetre scale. Previously, a harvesting mechanism through acoustic focusing was numerically simulated, where multilateral reflective metamaterials created an enclosed system that reflected sound energy onto a piezoelectric bimorph situated behind the sound source.^[^ ^17^ ^]^ The system was limited for practical implementation as the location of the sound source was restricted within the enclosure, but it demonstrated how focusing can powerfully amplify the captured energy and reduce losses. Here, we experimentally demonstrate the first strategy. By drawing inspiration from the concept of focusing, while leveraging the versatility of holography, we present a novel AEH system that projects the acoustic holographic output onto the harvester using a transmissive labyrinthine acoustic metamaterial (LAM). Sound energy, which is incident over a large area, would be concentrated with higher intensity by the LAM onto a smaller area. By situating the energy harvester within this smaller area, the harvesting efficiency is significantly improved.

With a single LAM, it is possible to propagate multiple focal points at various locations. This implies that the harvester does not have to be directly aligned with the original trajectory of input source waves, as the LAM can precisely channel the output elsewhere within a diffraction‐limited spatial plane. Our proposed approach for the first strategy provides the means for implementing and adapting the second strategy, where an array of focal points could be projected onto a large array of low‐cost piezoelectric receivers. Moreover, our holographic mechanism would allow the flexibility of harvesting selectivity, where different receivers could be activated based on the generated acoustic pattern. For example, for a 6 × 6 array of receivers, the metamaterial could project a pattern of 6 focal points in a horizontal line. Harvesters that fall within this line would receive higher acoustic energy compared to those not within that line.

In our study, we use different piezoelectric films, which are placed on the propagated sound target plane for enhanced power generation. We operate with airborne ultrasound at a lower frequency and demonstrate high experimental precision with our holographic approach using the LAM. This is further illustrated in Section 3.3. Furthermore, the harvester can be placed at a greater distance away from the sound source, unlike resonance‐type systems. Within a Helmholtz‐resonator AEH system, the harvester is often tightly coupled with a resonator unit in a 1:1 ratio. To scale the system over a larger area, the number of resonators must increase, along with a proportionate increase in the number of harvesters. In contrast, increasing the number of harvesters is optional for the LAM. The LAM may be extended over a larger area to capture a greater input, but the energy can be focused onto a smaller number of harvesters, which will potentially save cost. Additionally, LAMs are ultrathin, material agnostic, and easy to fabricate through additive manufacturing. The LAM also provides unprecedented potential for holographic projection energy harvesting as it is based on metamaterial technology that is reconfigurable. Memoli et al.^[^ ^23^ ^]^ and Cummer et al.^[^ ^24^ ^]^ reported labyrinthine units that can be reassembled on‐demand for different holographic projections.

While the LAM is fully scalable to audible frequencies, here, we experimentally implement AEH at 40 kHz. Ultrasound (>20 kHz) is useful for a broad range of applications, from haptic devices to nondestructive testing, imaging, or wireless charging of medical implants.^[^ ^25^, ^26^, ^27^, ^28^, ^29^, ^30^, ^31^ ^]^ In particular, emerging applications operating with ultrasound can benefit from the integration of efficient energy harvesting systems with the LAM. Examples include directional speakers,^[^ ^32^, ^33^ ^]^ Internet of Things devices or sensors^[^ ^34^ ^]^ in dashboards of cars, and haptic devices,^[^ ^35^ ^]^ which have become pervasive with virtual reality and augmented reality applications. These applications can operate within a 20–60 kHz frequency range.

In this work, we investigate the effectiveness of the LAM for ultrasonic energy harvesting. We study the responses of conventional commercial piezoelectric films as well as a lab‐made nanocomposite harvester film^[^ ^36^ ^]^ within the proposed AEH system. The films are confined to a small circular exposure area of 78.6 mm^2^, in comparison with the 27 722 mm^2^ LAM, which is over 350 times larger. The films are placed 30 cm away from the sound source to collect the focused energy. An observable gain (>150%) in both voltage output and power density demonstrates the precision and amplification capability of the experimental LAM, paving the way for integrating acoustic holography‐based mechanisms for efficient AEH in real‐world scenarios.

## 2 Design and Development

**Figure 1:**
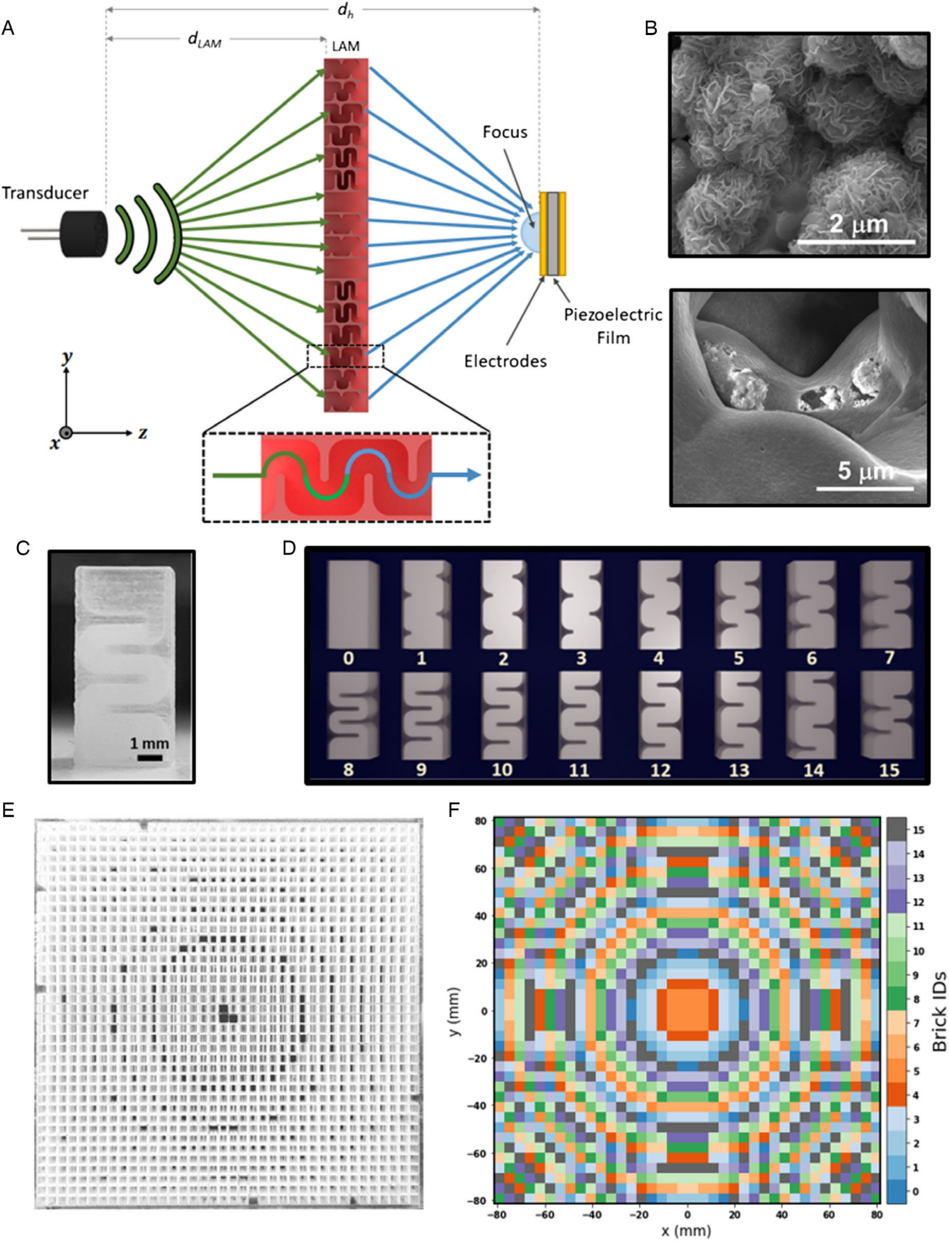
A) Schematic illustration of the concept of enhanced acoustic energy harvesting using a labyrinthine acoustic metamaterial (LAM). B) Scanning electron microscopy (SEM) microscopic image of MoS_2_ nanoflowers with particle sizes in 1–2 μm range (top), SEM image of nanocomposite harvester film, with MoS_2_ nanoflowers embedded PVDF film (bottom). C) Photograph of a single meta‐brick. D) Schematic illustration of all the 16 meta‐brick designs, each capable of providing individual amplitude and phase modulations. E) A fully printed single‐focus LAM composed of 38 × 38 meta‐bricks, and F) the corresponding geometrical description of the arrangement of meta‐bricks in the LAM to create a desired centralized focal point.

**Figure** **1**A schematically depicts our energy harvesting concept. An ultrasound (40 kHz) transducer source is placed at a distance (*d*_LAM_ = 20 cm) away from the LAM, which focuses the sound waves onto the surface of the piezoelectric energy harvester placed on the far side, with a focal length (10 cm). We fabricated MoS_2_ nanoflowers (Figure 1B) embedded PVDF harvester film, whose SEM image is shown in Figure 1C, which is elaborated further in Section 2.3. The following sections detail the computation and fabrication of the LAM and nanocomposite harvesting film.

### 2.1 Labyrinthine Acoustic Metamaterial

We designed and developed a planar transmissive LAM (166.5 mm × 166.5 mm × 8.66 mm), typically referred to as a metasurface,^[^ ^37^ ^]^ for modulating and patterning a 40 kHz ultrasonic wave. Typically, an acoustic metasurface consists of an array of repeating units arranged together to achieve the required functionality. Similarly, the LAM consisted of an arrangement of 16 distinct geometrical units,^[^ ^23^ ^]^ as shown in Figure 1D. These individual units were called metamaterial bricks (meta‐bricks). Each meta‐brick (4.33 mm × 4.33 mm × 8.66 mm) consisted of different space coiling elements that modulated the absolute path traveled by the sound waves, which provided a unique phase delay (ranging from −π to +π) to the output sound wave. These elements within the *meta*‐brick were subwavelength (i.e., less than the wavelength (λ ≈ 8.66 mm) of the operating frequency (40 kHz)) in dimension. For ultrasonic operation, our subwavelength meta‐bricks were fabricated with high‐resolution three‐dimensional (3D) printing.^[^ ^38^ ^]^ The LAM was printed with VeroClear plastic, which allowed it to be light and portable (see Experimental section). The elements within the meta‐brick and the position of each meta‐brick inside the LAM were chosen based on the required analog phase map built using the Gechberg–Saxton (GS) algorithm and piston model.^[^ ^23^ ^]^ Based on this computational design algorithm, the LAM's design could be reiterated to achieve different holographic patterns.

Figure 1E depicts a LAM designed for creating a single focal point of acoustic pressure. The LAM is scalable; for example, by increasing the size of the meta‐bricks by ten times to match a wavelength of 86.6 mm, LAM can be made to work for a lower frequency of 4 kHz while retaining the same functionalities.

### 2.2 Computational Metamaterial Design

The complex pressure $\psi_{t}$ of a transducer *t* is modeled as a point source with initial amplitude $a_{t}$ and phase $\varphi_{t}$ ^[^ ^39^ ^]^

(1) $$\psi_{t}=a_{t}\cdot e^{i\varphi_{t}}$$

A directivity function, describing a complex directional pattern of acoustic wave radiation emanating from a flat circular piston as it travels to strike a point *p* on the LAM surface, is formulated using the transducer piston model.^[^ ^40^, ^41^ ^]^

(2) $$\xi_{\text{t, p}}=\frac{2\cdot J_{1}\left(k\cdot (d/2)\cdot \text{sin}\theta \right)}{k\cdot (d/2)\cdot \text{sin}\theta }\cdot \frac{P_{\text{ref}}}{r_{t}}\cdot e^{ikr_{t}}$$

where $P_{\text{ref}}$ is the reference pressure of the transducer driven at maximum amplitude and measured at a distance of 1 m; *d* is the diameter of the transducer; *k* is the wavenumber; $r_{t}$ is the distance between the points *t* (transducer) and *p*; and *θ* is the angle between the vector $\bar{\text{tp}}$ connecting these points and the transducer normal, as shown in Figure S6, Supporting Information. Finally, $J_{1}$ is a Bessel function of the first kind.

The acoustic pressure field at a given point $p_{i,j}$ on the LAM surface is determined via the product of the transducer complex pressure $\psi_{t}$ and the directivity function $\xi_{t,p_{i,j}}$ calculated for that point.^[^ ^39^ ^]^

(3) $${\text{Ψ}}_{p_{i,j}}={\text{ψ}}_{t}\cdot \xi_{t,p_{i,j}}=a_{t}\cdot e^{i{\text{φ}}_{t}}\cdot \xi_{t,p_{i,j}}$$

The complex pressure generated by the transducer and incident on the all the points across the LAM surface ($\Psi_{\text{in}}$) is represented as a matrix.

(4) $$\Psi_{\text{in}}=\Psi_{t}\cdot \left[\begin{array}{ccc} \xi_{t,p_{0,0}} & \cdots & \xi_{t,p_{0,n}}\\ \vdots & \ddots & \vdots \\ \xi_{t,p_{m\text{, 0 }}} & \cdots & \xi_{t,p_{m,n}} \end{array}\right]=a_{\text{in}}\cdot e^{i{\text{φ}}_{\text{in}}}$$

where the subscripts of *p* refer to the coordinates of each point at the center of each element in the LAM surface. As in Equation (1), the incident complex pressure field can also be represented as incident amplitude ($a_{\text{in}}$) and phase ($\varphi_{\text{in}}$) matrices. After obtaining the complex pressure distribution over the LAM surface, we calculate the map of phase delays that the metamaterial must provide to generate a desired pattern. For this, we use either the Gerchberg–Saxton (GS) phase retrieval algorithm^[^ ^42^ ^]^ for double focus or simple geometric methods^[^ ^43^ ^]^ for a single focus (Further details in Section1.1, Supporting Information**)**. We represent the phase delay map as the matrix $\varphi_{\text{del}}$. However, since GS provides continuous phase values, we discretize $\varphi_{\text{del}}$ into 16 phase bins, corresponding to the 16 distinct metamaterial bricks, with evenly spaced values in the interval 0 to 2*π* (as shown in Figure 1F). The discretized phase delay map is represented by the matrix φdel'. We sum the incident phase matrix and the discretized phase delay matrix in the complex domain to get the total complex pressure field distribution on the surface of the LAM as

(5) $${\text{ψ}}_{\text{LAM}}\text{= }\left|{\text{ψ}}_{\text{in}}\right|\times e^{i\left({\text{φ}}_{\text{in}}{+\;\varphi }_{\text{del}}\prime \right)}$$

To simulate the complex pressure matrix at the harvesting plane (${\text{ψ}}_{z}$), we propagate this total complex pressure using the angular spectrum method.^[^ ^44^ ^]^

(6) $$\Psi_{z}=\text{IFT}[\text{FT}[\;\Psi_{\text{LAM}}]e^{i\sqrt{k^{2}-k_{x}^{2}-k_{y}^{2}}\cdot (d_{h}-d_{\text{LAM}})}]$$

Here, FT and IFT refer to the Fourier transform and inverse Fourier transform, respectively, $k_{x}$ and $k_{y}$ are the conjugate variables of the wavenumber *k* in the *x* and *y* directions, and $d_{h}$ and $d_{\text{LAM}}$ are the normal distances of the harvesting and LAM planes from the origin, respectively, as shown in Figure 1A.

### 2.3 Nanocomposite Harvester Film

A nanocomposite harvester film was fabricated by embedding hydrothermally grown piezoelectric molybdenum sulfide (MoS_2_) nanoflowers inside a PVDF polymer film as described in previous work.^[^ ^36^ ^]^ Interfacial interactions of the MoS_2_ with PVDF functional groups led to the rearrangement of PVDF chains into the electroactive β phase, which rendered the whole film piezoelectric. After doping inside the PVDF, the MoS_2_ nanoflowers induced spherulite formation characteristic of the β phase. Figure 1B shows the electron microscopy image of MoS_2_ nanoflowers with particle sizes in the 1–2 μm range, and Figure 1C shows the microscope image after doping these nanoflowers in PVDF. The thin piezoelectric film (≈40 μm) was packaged with copper electrodes on both sides for energy measurements (Figure S1, Supporting Information).

## 3 Results

Each acoustic energy harvester film was exposed to ultrasonic waves over a fixed circular focal area (78.6 mm^2^) for optimal performance. An initial characterization was performed to obtain the exact position and spread of the focal point of the LAM. Thereafter, the film was positioned for measurement with and without the ultrasonic field to determine the output voltage through an oscilloscope. Subsequently, the LAM was employed to focus the ultrasound on the harvester film, and the corresponding gain in output voltage and output power was determined.

### 3.1 Characterization of LAM by Acoustic Pressure Measurements

**Figure 2:**
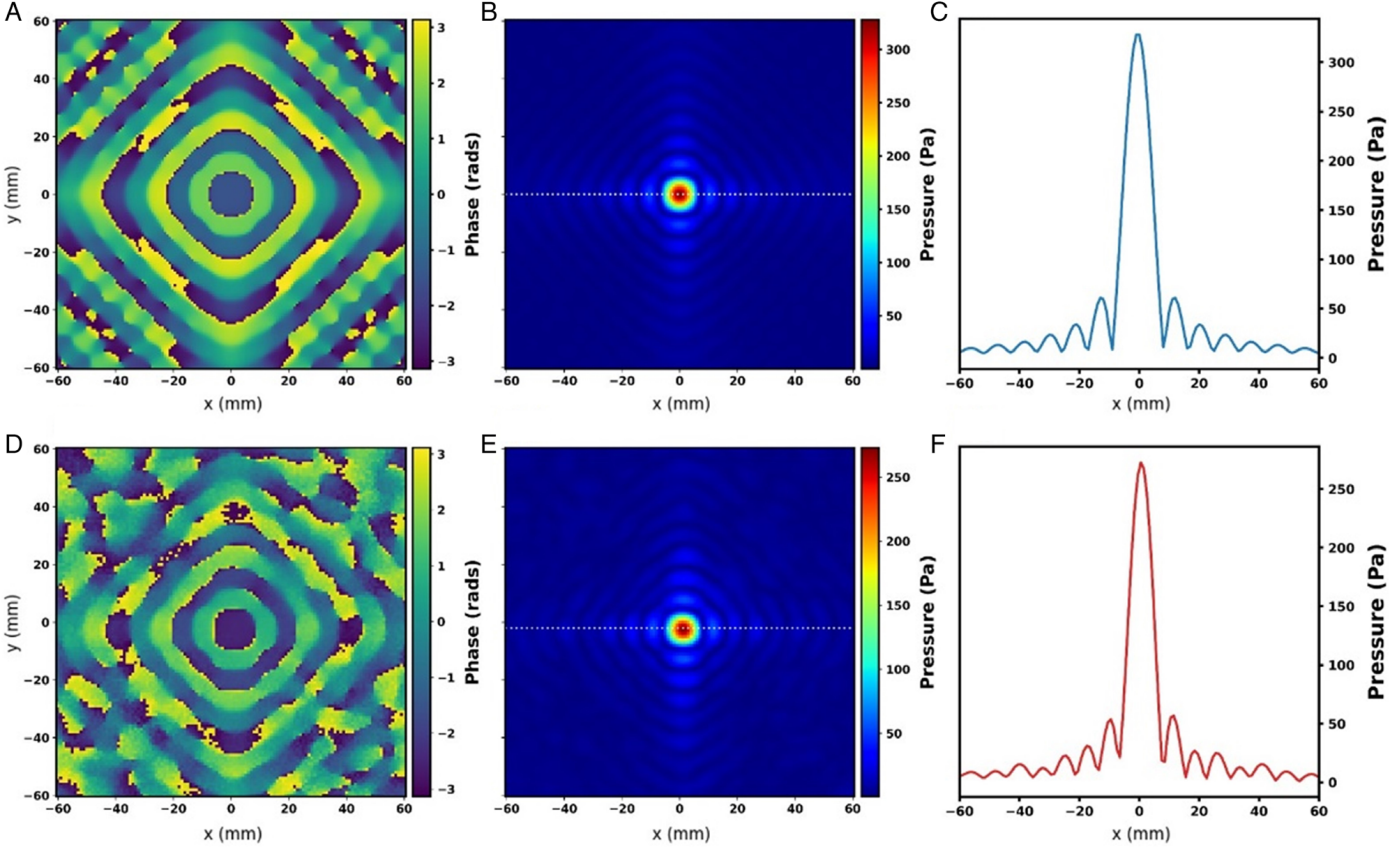
The simulated A–C) and experimentally measured D–F) acoustic phase (left) and pressure maps (middle) and line plot for acoustic pressure (right) for 40 kHz ultrasound wave, captured at an x‐y plane (such that the LAM center is at the center of *x*‐axis and *y*‐axis here, i.e., (0, 0) mm position). The cutline for the plots (C,F) is shown in pressure maps (B) and (E), respectively, marked with a black dotted line. The acoustic pressure is in Pascal (Pa), and the phase map varies from −π to +π.

Before using the LAM for harvesting, we first conducted acoustic pressure measurements without the harvester film to test and characterize the LAM for generating acoustic focusing. The experimental setup **(**Supplementary Figure S2, Supporting Information) consisted of an ultrasound source (transducer), a microphone, and a picoscope with an amplifier to record the acoustic pressure output (see Experimental section). The acoustic pressure amplitude and phase measurements were performed for a LAM designed to create a single focus. The LAM focused the sound at the desired focal point, and the microphone, which was attached to a computer numerical control (CNC) arm, scanned the focal plane around the center of the LAM with an area of 120 mm × 120 mm and step size of 1 mm. All the acoustic pressure measurements showed good agreement with the numerical computation, as shown in **Figure** **2**. Our simulations were based on the holographic techniques like the Gechberg–Saxton algorithm to create focal points and acoustic patterns using metamaterials. In comparison to a real‐world scenario, our simulation assumed an anechoic chamber with no reflections from the surrounding and no temperature variations. With the LAM, the maximum measured pressure at the focal point was 272.5 Pa (Figure 2F), while the maximum pressure for a transducer without a LAM (Figure S4, Supporting Information) was only 20 Pa. This shows that a 13.6 times pressure gain was achieved using the LAM to focus the ultrasound. The simulated focal point had a pressure of 328 Pa. The experimentally measured values were lower than the simulated data. The discrepancies between the experiments and simulations were attributed to the reflections from experimental setup, table, walls, etc. and other environmental losses, which were not accounted for in the simulation.

### 3.2 Energy Harvesting Measurements

The experimental setup followed the schematic in Figure 1A. We 3D‐printed support structures to support and align the LAM and harvester film vertically (as shown in Figure S3, Supporting Information). The harvester film was taut and clamped to ensure that a fixed surface area of the film (78.6 mm^2^) was exposed to the ultrasonic waves.

#### 3.2.1 Voltage Gain

**Figure 3:**
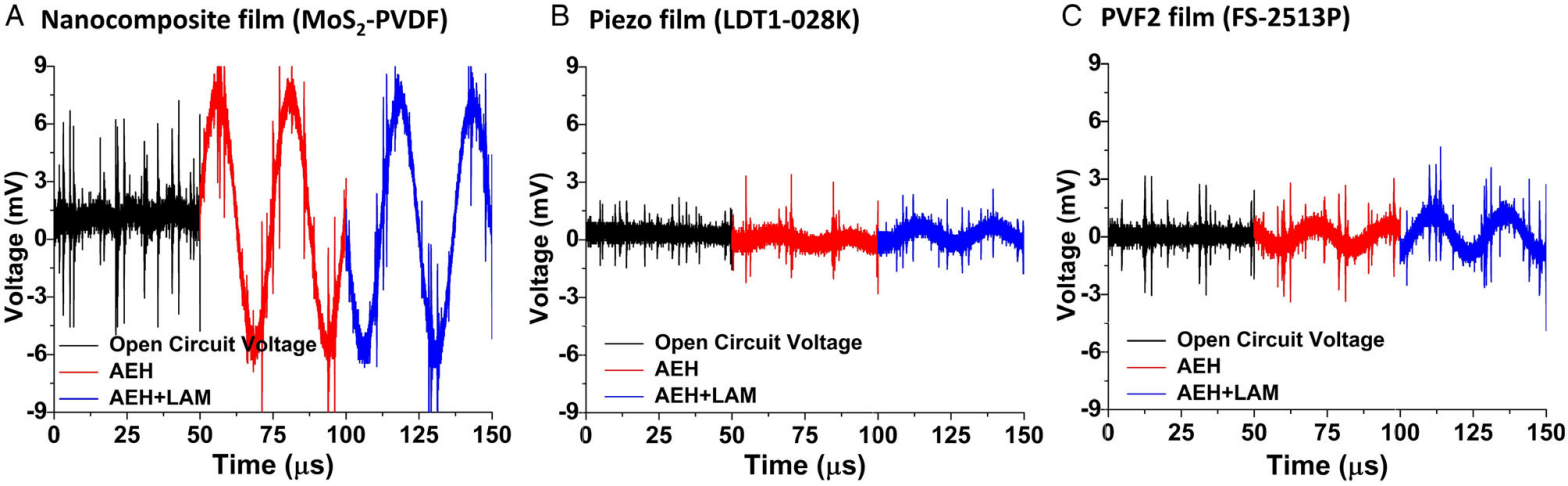
Output voltage for an open circuit (black), unfocused ultrasound pressure (Transducer ON) during normal AEH (red), and focused ultrasound for AEH with LAM (blue) for the three harvester films, A) nanocomposite harvester film, B) commercially available piezo film (LDT1‐028 K), and C) polyvinylidene fluoride (PVF2) film (FS‐2513P).

We used three energy harvester films to investigate the efficiency of the proposed approach of using the LAM for focusing sound energy. The three films were 1) fabricated nanocomposite MoS_2_ embedded PVDF harvester film; 2) commercially available piezo film (LDT1‐028K sensor); and 3) commercially available polyvinylidene fluoride (PVF2) film (PROWAVE FS‐2513P Sensor Piezo Film). An oscilloscope (KEYSIGHT InfiniiVision DSO‐X 3024A) was used to capture the output voltage from the harvester films at a sampling rate of 312.5 kSa s^−1^ to 0.5 GSa s^−1^. The output voltage signals captured from the oscilloscope for the three harvester films are plotted in **Figure** **3**. These voltage signals were captured for three cases involving 1) an open circuit (black); 2) spread ultrasound pressure (Transducer ON) during normal AEH (red); and 4) focused ultrasound pressure using LAM for AEH (blue). The focused ultrasound using the LAM showed a larger voltage output compared to the unfocused ultrasound for all the commercial films. The period of the signal was found to be 25 μs, implying a signal frequency of 40 kHz, which is equivalent to the provided ultrasound signal.

The Young's modulus of the nanocomposite MoS_2_‐PVDF film was found to be 928 × 10^6^ N m^−2^, which is much lower than the Young's modulus of the commercial LDT1‐028K piezoelectric film i.e. 2.4 × 10^9^^[^ ^45^, ^46^ ^]^ and 2 × 10^9^ N m^−^^2^ for the polyvinylidene fluoride (PVF2) film (FS‐2513P).^[^ ^47^ ^]^ The piezoelectric coefficient of the nanocomposite film was found to be 36.4 pC N^−1^, and it is larger than 24 pC N^−1^, which was reported for commercial PVDF films.^[^ ^36^, ^47^ ^]^ These piezoelectric coefficients determined the electromechanical coupling factor of the piezoelectric energy harvester structure that provided a reason for the higher mechanical‐electrical energy conversion response of the nanocomposite film toward the ultrasonic field than the piezoelectric commercial films.

**Figure 4:**
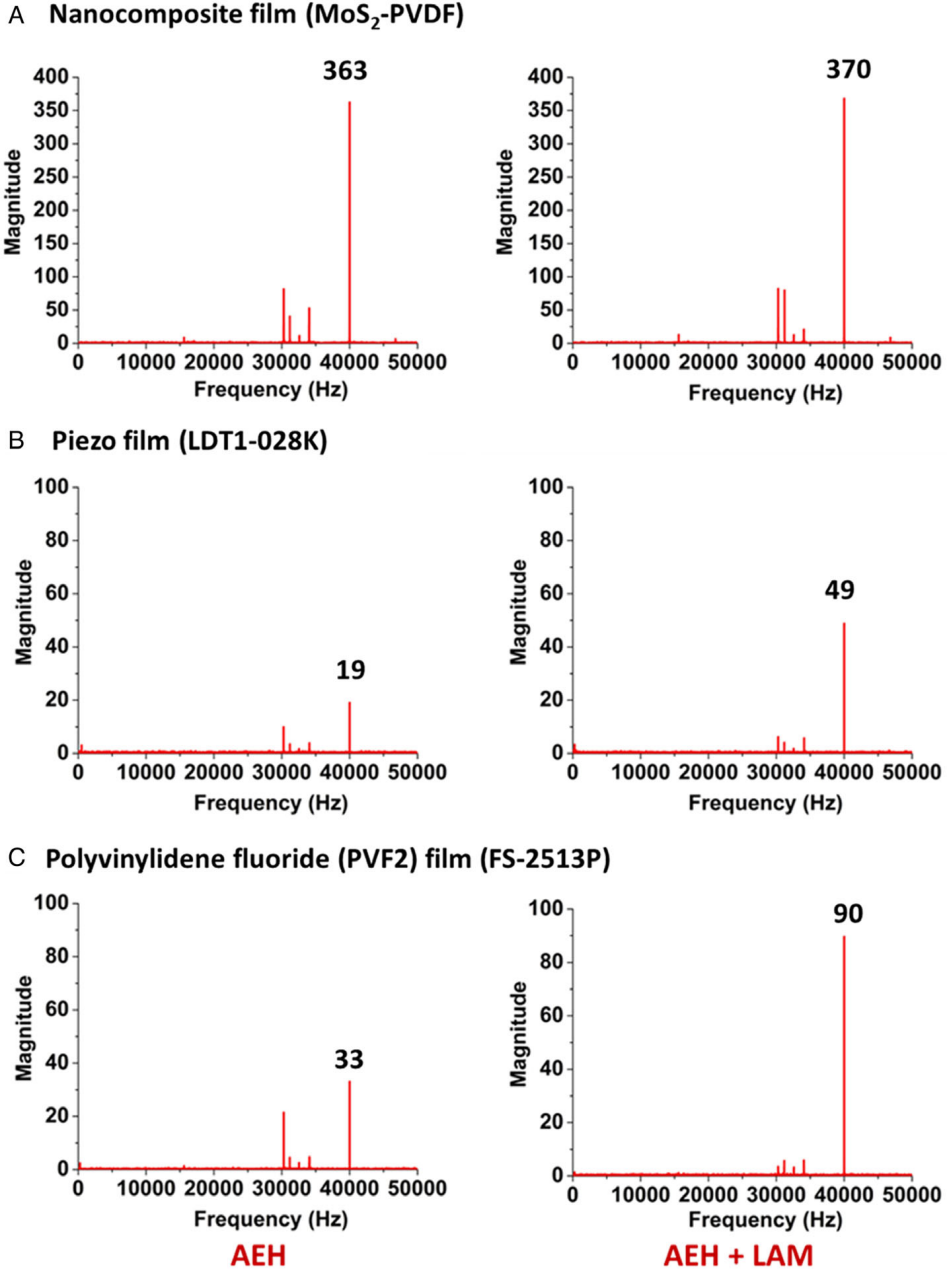
Plots showing magnitude against frequency, obtained from the FFT of the output voltage signals for the A) nanocomposite piezoelectric harvester film, B) commercial piezo film (LDT1‐028K sensor), and C) PVF2 film (FS‐2513P) under differently modulated ultrasonic forces, i.e., unfocused ultrasound for AEH (left), and focused ultrasound by using LAM with AEH (right).

To quantify the gain in 40 kHz frequency voltage output using focused ultrasound with the LAM, we captured the output signal at a sampling rate of 312.5 kSa s^−1^ and implemented a Fast Fourier Transform (FFT). The Fourier transform magnitude is shown in **Figure** **4**. We removed the open circuit voltage noise from the computed signals. The peak magnitude values at 40 kHz obtained from the FFT of output voltage signals for AEH with and without the LAM for the three harvester films are tabulated in **Table** **1**. It was found that the fabricated nanocomposite harvester film was extremely responsive to the ultrasonic field with a magnitude greater than 300, which was significantly larger than the magnitude of other commercial films, which were less than 100. The frequency band of transducer varied from 30 to 50 kHz (the resonance is at 40 kHz); thus, we observed smaller peaks within this frequency range in the FFT in Figure 4. We also calculated the percentage gain in the magnitude by using the LAM compared to the case when there was no LAM. We found that the commercial films showed a significant gain of 157–173% by using focused ultrasound through the LAM over a similar signal without the LAM. The nanocomposite film did not show a comparable gain (1.92%) to the commercial films using focused ultrasound through the LAM, which could be attributed to some unknown phenomenon like the molecular interaction of molybdenum sulfide nanoflowers with ultrasound, non‐linear self‐poling characteristics of the film, saturation, or some other effect which is not understood as a part of this work and has scope for future investigation.

**Table 1:** Peak magnitude, obtained from the FFT of voltage signals at 40 kHz frequency from different harvester films, with and without the LAM

	AEH	AEH + LAM	Gain [%]
Nanocomposite film	363	370	1.92
Piezoelectric film (LDT1‐028K)	19	49	157.89
Polyvinylidene fluoride (PVF2) film (FS‐2513P)	33	90	172.72

#### 3.2.2 Power Gain

**Figure 5:**
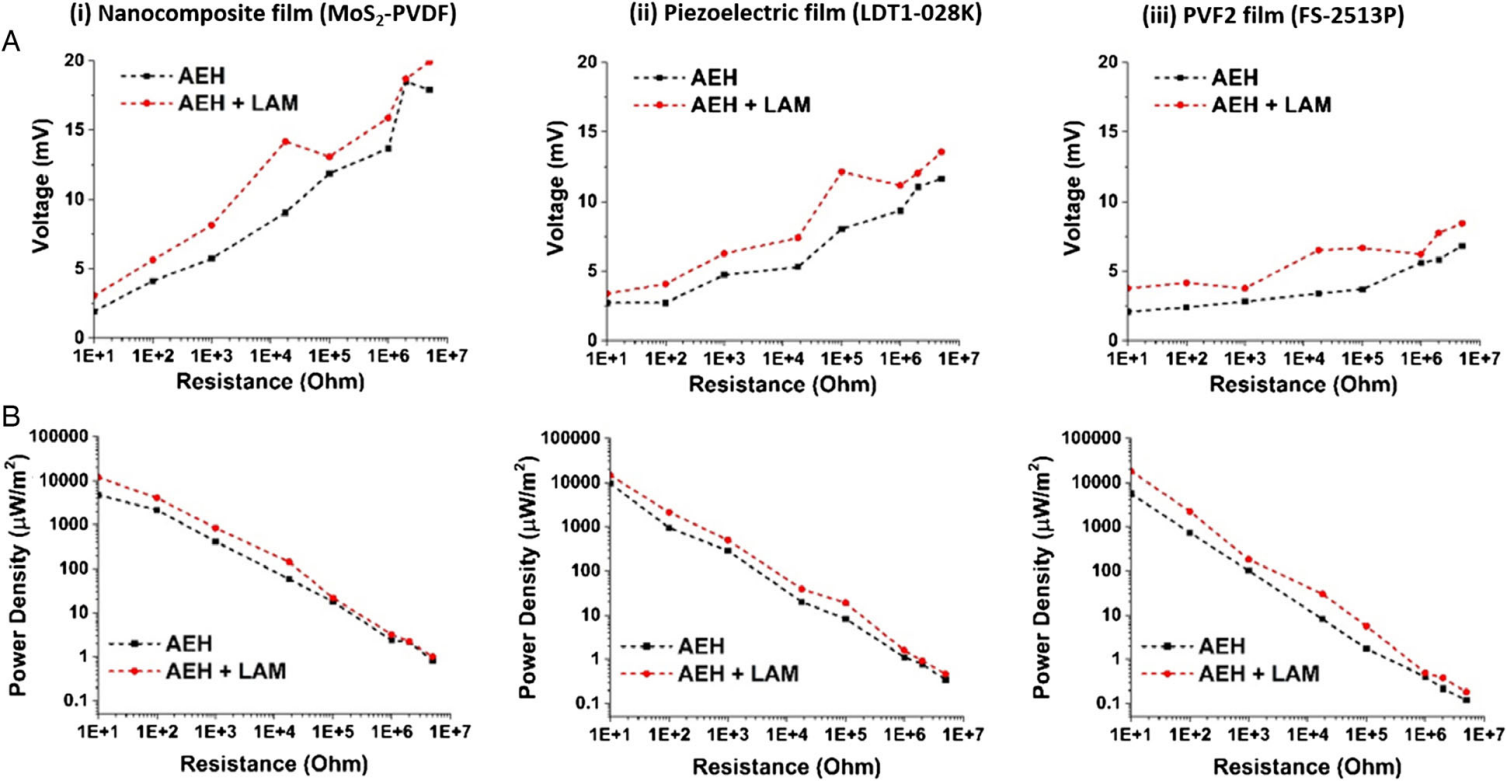
A) Output voltage and B) output power density of the three piezoelectric harvester films, namely, 1) nanocomposite MoS_2_ embedded PVDF harvester film; 2) piezo film (LDT1‐028 K); and 3) polyvinylidene fluoride (PVF2) film (FS‐2513P), with respect to the load resistances for harvesting acoustic energy with and without the LAM.

We also investigated the power generated by all the piezoelectric harvester films with and without the LAM by connecting different load resistances from 10 Ω to 5 MΩ across the circuit and capturing it through an oscilloscope (sampling rate 125 MSa s^−1^) (shown in Figure S5, Supporting Information). We observed that the output voltage increased with an increase in resistance and by using LAM for focusing the ultrasound for all the films, as shown in **Figure** **5**A. Further, we determined the power as $=\frac{V^{2}}{R_{L}}$ across a circular area of a 5 mm radius. The maximum power density was found to be 18.2 mW m^−2^ for the PVF2 film (FS‐2513P). The power density for all the films decreased with an increase in resistance, but the power density was always higher for LAM‐based AEH as compared to the case without the LAM, thus proving our hypothesis of achieving a higher harvesting efficiency using the LAM (shown in Figure 5B). The maximum power density gain was found to be 272% for the PVF2 film (FS‐2513P) at an 18 kΩ resistance over a similar system without a LAM.

### 3.3 Multipoint Focusing

**Figure 6:**
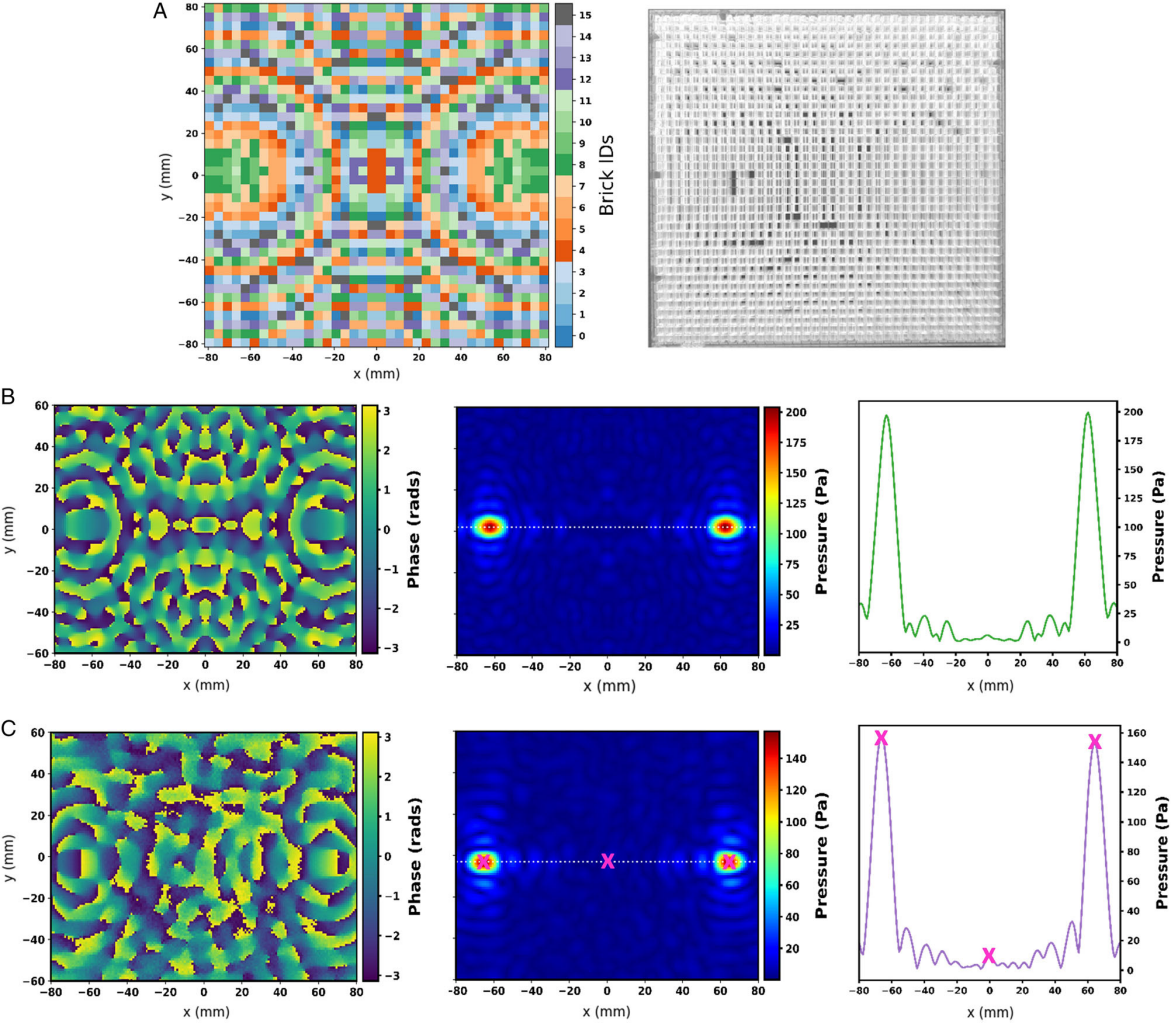
A) Computed brick IDs (left) and the associated LAM's experimental image (right) for a geometrical description of a double focus LAM. B,C) show the simulated and experimentally measured acoustic phase (left) and acoustic pressure maps (middle) and a line plot for acoustic pressure (right) for a 40 kHz ultrasound wave, captured in an *x*–*y* plane (such that the LAM center is at the center of the *x*‐axis and y‐axis here, i.e., (0, 0) mm position). The cutline for the line plots in B,C) is shown in corresponding pressure maps (middle) marked with a white dotted line. The acoustic pressure is in Pascal (Pa), and the phase map varies from −π to +π.

To demonstrate the ability of LAM for simultaneous AEH at multiple targeted locations, we designed another LAM device (as shown in **Figure** **6**A **)** to achieve more than one focal point. This LAM was designed to create two symmetrical focal points with a spacing of 120 mm in the same focal plane as a single‐focus case, which is 10 cm away from the LAM and 30 cm away from the transducer. By splitting the focal points, we aimed to cover a larger surface area on the exposed film. We scanned the target plane around the center of LAM with an area of 160 mm × 120 mm and a step size of 1 mm to measure the acoustic pressure. We observed that the maximum sound pressure on a given point decreased with an increase in the number of focal points. The maximum pressure was 272.5 Pa for a single focal point, while it decreased to 157 Pa for two symmetrical focal points. Figure 6B,C shows the acoustic pressure measurements and acoustic phase maps calculated by numerical computation and experimentally, respectively, for two focal points. Although the incident absolute pressure amplitude decreased (nearly to half of the single‐focus case) for the double focus, the exposed surface area on the harvester film increased (double of the single focus). Thus, overall, the harvesting efficiency of a double focal point LAM remained the same as the single‐focus LAM. In Figure 6C, along the white dotted line in the pressure plot, we included three pink markers to illustrate the potential for selective harvesting through our holographic mechanism. For instance, we could place three piezoelectric receivers at the positions of these markers. Two receivers would be centered at the two focal points, respectively, and the third placed at the center without any focused pressure. According to the line plot, it is clear that the position at the center marker would receive much lower pressure than the ones on the sides. This difference would be approximately 10‐fold. Future research can explore the implementation of novel holographic metamaterials for different functionalities like creating multiple focal points positioned asymmetrically on the harvester film or focusing sound behind an obstacle to achieve AEH with an efficient power gain.

## 4 Conclusion

We present a novel approach for enhancing the efficiency of AEH by exploiting the distinctive properties of the specially designed LAM. The transmissive LAM has unit cells called meta‐bricks with internal geometries that take the form of a space coiling pathway through which the sound travels, and its path length determines the acoustic phase modulation between −π and +π. The LAM does not only capture the incident sound over a wide area but can also shape the divergent acoustic field to converge to a focal point, thus realizing a 252.5 Pa acoustic pressure gain (13.6 times more by using the LAM). We measure the variation in harvesting efficiency using two commercial piezoelectric films and a nanocomposite harvester film made by inculcating MoS_2_ nanoflowers in a PVDF matrix film. We quantify a 157–173% voltage gain on the commercial piezoelectric harvester films by using the LAM as compared to an AEH system without the LAM, while the nanocomposite harvester film shows a lower gain of 1–2%. We obtain a high maximum power density value of 18.2 mW m^−2^ and a maximum power gain of 272%, using the LAM with the PVF2 harvester film over a similar system without a LAM. We also utilize the GS algorithm, a holographic phase retrieval technique, for creating multipoint focusing. For future work, new LAM designs can be implemented to generate different high‐intensity acoustic holographic patterns to maximize sound collection and optimal target areas for harvesting, like focusing sound behind an obstacle and generating a LAM with different asymmetrical focal points etc., thereby enabling high‐yield AEH output in real‐world scenarios. This research opens the pathway to realize next‐generation AEH systems using AMMs for holographically patterning the sound to maximize sound capturing and harvesting efficiency, bringing us a step closer to embracing sound as a viable energy resource.

## 5 Experimental Section

5.1

5.1.1

##### Fabrication of Labyrinthine Acoustic Metamaterial (LAM)

LAMs were 3D‐printed as monoliths using the Stratasys J750 multimaterial Polyjet. Polyjet printing was employed for our application due to 1) print resolution; and 2) high build volume. Polyjet additive manufacturing technology has exceptionally high resolution compared to commercial fused‐deposition modeling printers, which were ideal for fabricating the meta‐bricks due to their small‐scale and complex internal geometries.^[^ ^48^ ^]^ The build envelope for the Polyjet is also large (490 × 390 × 200 mm), which was able to accommodate the size of our LAMs (base area, 166.5 mm × 166.5 mm = 27,722.25 mm^2^). Moreover, instead of printing in an opaque material, printing with the transparent VeroClear material provided better visibility of the geometries of the metamaterial units, which helped us to better identify any defects in the print. Due to the presence of overhangs, the meta‐bricks were printed with water‐soluble support material (SUP706), which was dissolved in a chemical bath solution of sodium hydroxide and sodium metasilicate. Support structures for the LAM and films were fabricated using fused‐deposition modeling (FDM) with the Ultimaker S5 and the Prusa i3, MK3S.

##### Transmission Field Mapping Measurements

Transmission amplitude was measured by mounting an acoustic source of a 40 kHz transducer (MA40S4S, Murata Electronics, Japan) at a distance of 20 cm from the LAM. The transducer has a beam spread angle of ±40° and sound pressure levels of 120 ± 3 dB (measured on the axis at *z* = 30 cm). The transducer was driven using a 20 *V*_pp_ square wave voltage signal with a 50% duty cycle at 40 kHz frequency, generated using a GW INSTEK AFG‐2225 Dual‐channel arbitrary function generator. Quantitative pressure measurements were obtained using a calibrated Brüel & Kjaer microphone (model 4138‐A‐015) positioned 10 cm away from the LAM. A 3D linear stage was built in our laboratory for scanning the 3D space. The signal received from the microphone was amplified using a mic preamp: Bruel & Kjaer 2670 and a 1‐channel Microphone Conditioning Amplifier 2690‐A‐0S1. The signals were sent to a PicoScope 4262 oscilloscope to record the differences between the received and reference input driving signals, which were used for calculating the phase of the acoustic waves. MATLAB and Python were used to plot the captured data for the acoustic pressure amplitude and phase maps. The transmitted normalized pressure was calculated as P(dB) = 20log(*P*_m_/*P*_0_), where *P*_m_ and *P*_0_ (10^−5^ Pa) are measured and reference acoustic pressures, respectively.

## Conflict of Interest

The authors declare no conflict of interest.

## Supporting information

Supplementary Material

## Data Availability

The data that support the findings of this study are available from the corresponding author upon reasonable request.
