# Supplementary material for: Transmissive Labyrinthine Acoustic Metamaterial‐Based Holography for Extraordinary Energy Harvesting
Source: Adv Eng Mater. 2022 Nov 9;25(4):2201117. doi: 10.1002/adem.202201117 (PMC10566638; doi:10.1002/adem.202201117)
Supplement: Supplementary file 1 — Supplementary Material [file ADEM-25-0-s001.pdf]

Supporting Information

Transmissive Labyrinthine Acoustic Metamaterial based Holography for Extraordinary Energy Harvesting

Shubhi Bansal, Christabel Choi, James Hardwick, Biswajoy Bagchi, Manish K. Tiwari, Sriram Subramanian*

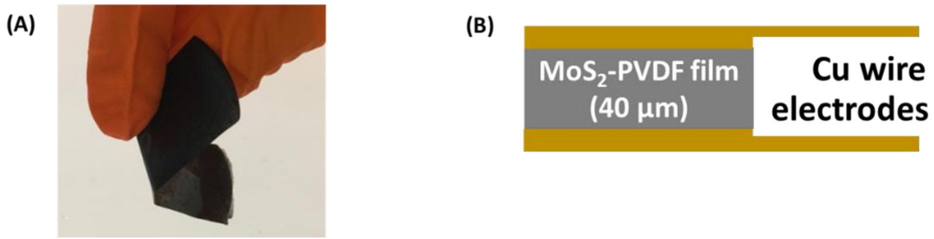

Figure S1. a) The photographic image of the MoS_2 embedded PVDF film, which is thin and flexible. b) Schematic of the composite energy harvester film with copper electrodes.

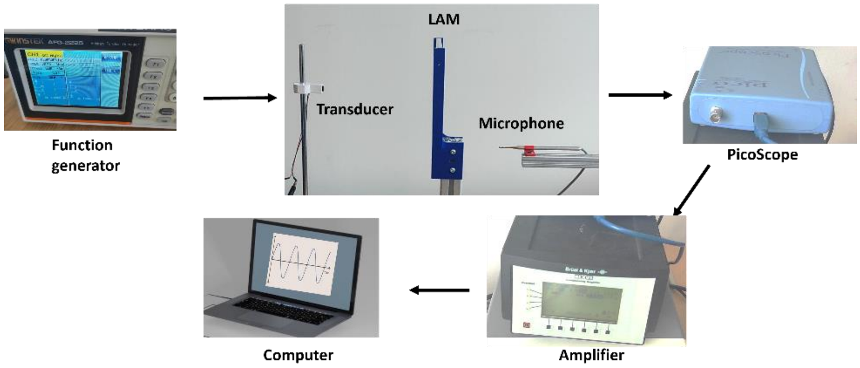

Figure S2. Schematic of the components of the experimental setup for acoustic pressure measurements using LAM.

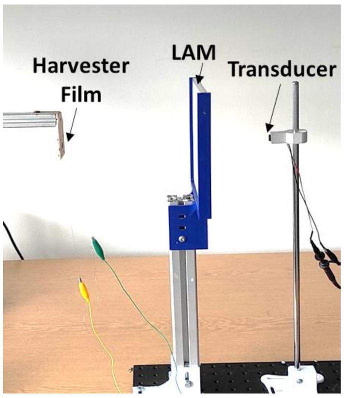

Figure S3. Photographic image of the experimental setup during energy harvesting measurements.

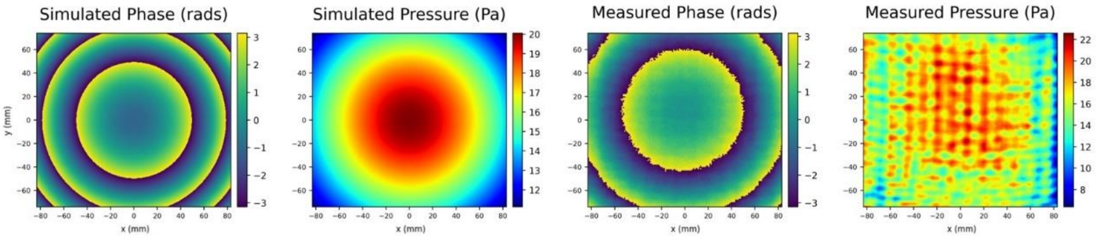

Figure S4. The (A) simulated and (B) experimentally measured acoustic phase (left) and acoustic pressure (right) maps for 40 kHz wave, captured at an x-y plane with the transducer placed at the centre of the x-axis and y-axis here, i.e. (0, 0) mm position. The absolute acoustic pressure is in Pascal (Pa), and the phase map varies from -π to +π.

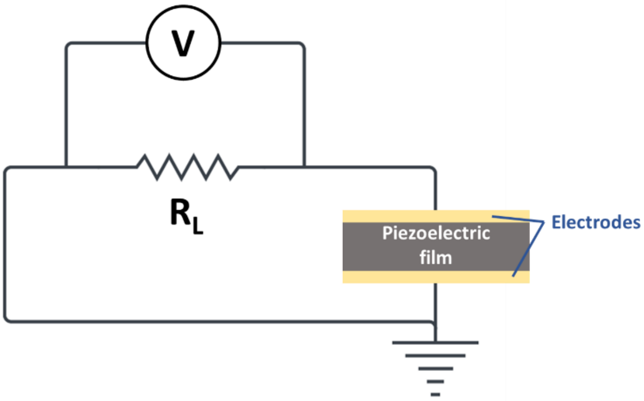

Figure S5. A schematic diagram of a piezoelectric harvester film with the load (R_L) and output voltage (V) read through an oscilloscope.

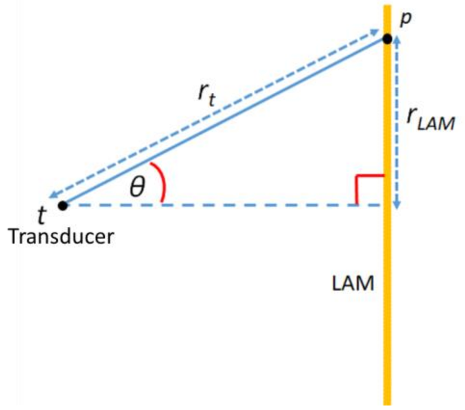

Figure S6. A schematic diagram of the LAM and transducer showing the parameters used in computational equations.

S1.1. Determining phase-delay maps of LAM

Depending on their functionality, i.e., single or double foci, we used two different methods to find the phase maps for the LAMs. In the case of a single focal point, a simple geometric approach was followed to find the phase map. Firstly, we determined the complex pressure that was produced by the transducer source and incident on the metamaterial surface using the piston model (Eqn 2). Secondly, we found the distance vectors between each element on the LAM surface and the focal point we wanted to generate. As the ultrasound (40 kHz) has a fixed wavelength (λ = 8.66 mm), we calculated how the phase of waves evolved as they travelled along each of the path vectors and thus, we determined their phase value when they arrived at the focus. Finally, we tuned each element to provide a phase delay such that all ultrasound waves arrived at the focus with the same phase and maximum superposition.

For a double foci LAM, a more complicated method of phase retrieval was used. This was achieved via the use of an iterative algorithm called the Gerchberg-Saxton (GS) algorithm[42]. This algorithm was originally devised for the optical regime but subsequently also used in acoustic problems. The GS algorithm is agnostic to the acoustic holographic image being generated and can be applied for any arbitrary glyph, character, or pattern within a certain resolution limit, which is directly informed by the surface area of the AMM.

The GS algorithm uses Fourier transforms to propagate the 2D complex pressure fields backwards and forwards between two planes, constraining and refining the pressure over the desired area increasingly as it iterates. We used ASM (Eqn 4) to propagate acoustic pressure between planes throughout the following process:

(1) We began with a target image of the acoustic holographic pattern to be generated at the target plane (z2). We back propagated this target image onto the metasurface plane z1 using ASM.
(2) We isolated this back propagated pressure over the area covered by the metasurface on plane z1 (we call this area the aperture). The phase over the aperture was saved, but the amplitude values were reset to the reference as determined by the acoustic wave source, as our metasurface modulates phase only.
(3) We then forward propagated the complex pressure isolated over the aperture back to the target plane z2 using ASM.
(4) We isolated the complex acoustic pressure over the target area in plane z2 by overlaying the target image onto this pressure distribution, resetting the values outside this area. All other amplitude and phase information at the target plane was discarded.
(5) Having completed one iteration of the algorithm, we obtained an updated version of our original target image at the target plane. We repeated the algorithm from steps 1-4, feeding the updated version as the new target until a satisfactory propagation at the target plane was returned. The last step, after the iterations were complete, was to account for the phase of the source waves incident on the metasurface before reflection and output the phase map on the metasurface.

The algorithm can be run for as many iterations as required to achieve a high-quality output image, although we asymptote to a steady solution after 100 to 200 iterations.
